# Expanding opportunities for chronic disease prevention for Hispanics: the Better Together REACH program in Pennsylvania

**DOI:** 10.3389/fpubh.2023.1134044

**Published:** 2023-06-20

**Authors:** William A. Calo, Betsy Aumiller, Andrea Murray, Laurie Crawford, Madeline Bermudez, Lisa Weaver, Maria Paula Henao, Nicole Maurer Gray, Vicki DeLoatch, Darilyn Rivera-Collazo, Janelle Gomez, Jennifer L. Kraschnewski

**Affiliations:** ^1^Department of Public Health Sciences, Penn State College of Medicine, Hershey, PA, United States; ^2^Department of Medicine, Penn State College of Medicine, Hershey, PA, United States; ^3^Penn State Health St. Joseph, Reading, PA, United States; ^4^Penn State Berks, Reading, PA, United States; ^5^Community Health Council of Lebanon County, Lebanon, PA, United States; ^6^Lebanon Family Health Services, Lebanon, PA, United States

**Keywords:** chronic disease prevention, Hispanics, physical activity, healthy eating, breastfeeding education, diabetes prevention program (DPP), health equity, community coalitions

## Abstract

**Background:**

Hispanics in Lebanon and Reading, Pennsylvania, experience high levels of socioeconomic and health disparities in risk factors for chronic disease. In 2018, our community-academic coalition “Better Together” received a Racial and Ethnic Approaches to Community Health (REACH) award to improve healthy lifestyles. This report describes our work-in-progress and lessons learned to date from our REACH-supported initiatives in Lebanon and Reading.

**Methods:**

For the past 4 years, our coalition has leveraged strong community collaborations to implement and evaluate culturally-tailored practice- and evidence-based activities aimed at increasing physical activity, healthy nutrition, and community-clinical linkages. This community case report summarizes the context where our overall program was implemented, including the priority population, target geographical area, socioeconomic and health disparities data, community-academic coalition, conceptual model, and details the progress of the Better Together initiative in the two communities impacted.

**Results:**

To improve physical activity, we are: (1) creating new and enhancing existing trails connecting everyday destinations through city redesigning and master planning, (2) promoting outdoor physical activity, (3) increasing awareness of community resources for chronic disease prevention, and (4) supporting access to bikes for youth and families. To improve nutrition, we are: (1) expanding access to locally-grown fresh fruit and vegetables in community and clinical settings, through the Farmers Market Nutrition Program to beneficiaries of the Women, Infants, and Children (WIC) program and the Veggie Rx to patients who are at risk for or have diabetes, and (2) providing bilingual breastfeeding education. To enhance community-clinical linkages, we are training bilingual community health workers to connect at-risk individuals with diabetes prevention programs.

**Conclusions:**

Intervening in areas facing high chronic disease health disparities leads us to develop a community-collaborative blueprint that can be replicated across Hispanic communities in Pennsylvania and the United States.

## Introduction

Hispanics are the largest racial minority group in the United States (US). In 2020, about 61 million people of Hispanic origin were living in the country (1). By 2060, it is projected that the Hispanic population will increase to over 111 million people, a significant factor in U.S. population growth (2). Hispanics experience high prevalence of most chronic diseases (3). For example, the prevalence of diabetes among Hispanic adults is 22.6% vs. 11.3% in non-Hispanic Whites (4). Risk factors such as obesity, sedentary behavior, and poor healthy eating, are a major concern for Hispanics and significantly contribute to chronic diseases like type 2 diabetes. Hispanic adults (47.0%) and youth aged 2–19 years (25.8%) have the highest prevalence of obesity in the US (vs. 37.9% and 14.1% in non-Hispanic Whites, respectively) (5). Hispanics are typically less likely to be aware of their conditions, receive instructions from their physicians to adopt lifestyle modifications, or self-monitor their conditions (6). If Hispanics continue to experience poorer chronic disease outcomes, the projected demographic change in the U.S. population will magnify these health inequities.

Pennsylvania is home to 1 million Hispanics (7). Vibrant Hispanic communities can be found in mid-size cities and towns like Lebanon (pop. 26,914; 44% Hispanic) in Lebanon County, and Reading (pop. 95,112; 67% Hispanic) in Berks County (7). Hispanics in both communities experience high levels of known socioeconomic factors associated with increased rates of chronic diseases (8–10). In particular, more than one-third (38.8%-46.0%) of Hispanics live below the poverty line, over 15% points above the U.S. average, and between 13.6% and 25.2% in the target communities had limited English proficiency compared to only 8.5% in the US and 4.1% in Pennsylvania (Table 1). Several community health needs assessments (CHNAs) conducted in the region, including Hispanic and non-Hispanic populations (11–14). prioritize chronic disease, obesity, sedentary behavior, and inadequate nutrition as health topics requiring immediate action. For example, adults in Lebanon reported higher percentages of inadequate fruit and vegetable consumption, heart disease, high blood pleasure, and cholesterol than the corresponding state and national averages (Table 1). Both communities also reported high percentages of adults with obesity and high rates for heart disease and cancer deaths. The size and rapid growth of the Hispanic population in these two communities, coupled with their high rates of socioeconomic disparities and chronic disease, offers considerable reason to focus on this population's chronic disease prevention and management.

**Table 1:** Socioeconomic and chronic disease health disparities in Lebanon and Reading.

	**Lebanon**	**Reading**	**Pennsylvania**	**US**
**Socioeconomic factors** ^*^				
Total population, *n*	25,654	87,899	n/a	n/a
Hispanic population, %	40.4	63.1	7.0	17.3
Hispanic population, *n*	10,374	55,508	n/a	n/a
Total population living below poverty, %	***25.7***	***39.2***	13.3	15.1
Hispanic population living below poverty, %	***38.8***	***46.0***	31.5	23.4
Limited English proficiency, %^1^	***13.6***	***25.2***	4.1	8.5
Hispanic population with no high school, %	***40.0***	***48.2***	30.7	34.2
Hispanic population without health insurance, %	17.2	*19.1*	17.4	23.4
Children eligible for free or reduced price lunch, %	***100***	***100***	48.1	52.6
Hispanic households receiving SNAP benefits, %^2^	***60.2***	***55.9***	36.0	22.3
Unemployment rate^3^	***4.7***	***5.1***	4.6	4.2
**Chronic diseases & risk factors** ^**^				
Inadequate fruit/vegetable consumption, %^4^	***78.5***	69.6	75.5	75.7
Population with no leisure time physical activity, %^4^	21.3	***22.2***	22.0	21.8
Soda expenditure^5^	*3.8*	***3.7***	3.5	4.0
Medicare population with diabetes, %^6^	25.3	*25.9*	25.8	26.5
Adults with heart disease, %^4^	***7.9***	2.8	5.1	4.4
Adults with high blood pressure, %^4^	***28.6***	24.8	27.2	28.1
Adults with high cholesterol, %^4^	***42.2***	32.6	37.9	38.5
Adults with obesity, %^4^	***29.1***	***31.9***	29.0	27.5
Adults overweight, %^4^	35.0	***36.4***	35.9	35.8
Heart disease age-adjusted death rate^7^	***169.5***	***169.2***	114.4	168.2
Cancer age-adjusted death rate^7^	***166.8***	***162.2***	109.1	160.9

In bold are disparities compared to the US, italicized are disparities compared to Pennsylvania, and bold and italicized are disparities compared to both Pennsylvania and the US. ^*^Data source is the U.S. Census Bureau American Community Survey 2012-16 (5-year estimates) unless otherwise noted and accessed via the Community Commons website. ^**^Some data reported at the county level because area estimates for the cities of Lebanon and Reading were not available or suppressed, and accessed via the Community Commons website. n/a = not applicable. ^1^Percentage of the population aged 5 and older who speak a language other than English at home and speak English less than “very well”; ^2^Supplemental Nutrition Assistance Program; ^3^Data source is U.S. Department of Labor, Bureau of Labor Statistics (March 2018) and accessed via the Community Commons website. ^4^Behavioral Risk Factor Surveillance System; ^5^Nielsen Site Reports, 2014; ^6^Centers for Medicare and Medicaid Services, 2015; ^7^National Vital Statistics System, 2012-16.

Established in 1999, Racial and Ethnic Approaches to Community Health (REACH) is the cornerstone program aimed at reducing racial and ethnic health disparities at the Centers for Disease Control and Prevention (CDC). In 2018, CDC funded a new 5-year cycle of grant recipients to reduce health disparities among racial and ethnic populations (e.g., Hispanics) with high levels of chronic disease (e.g., diabetes) (15). Through REACH, recipients and their community partners plan and carry out local, culturally appropriate programs to address preventable risk behaviors leading to chronic diseases (e.g., poor nutrition, physical inactivity) in socioeconomic deprived communities. Since 2018, Better Together REACH, our initiative, has leveraged strong community collaborations to implement locally-tailored practice- and evidence-based strategies aimed at increasing physical activity opportunities, healthy nutrition programming, and diabetes prevention programs in Lebanon and Berks counties. The overall objective of this report is to describe the work-in-progress and lessons learned from our REACH-supported initiatives. In particular, we describe the implementation activities and initial results of three strategies to improve physical activity (City redesigning and master planning, Nature Rx, and Lebanon Bicycle Recycle), three strategies to improve healthy eating (Farmers Market Nutrition Program, Veggie Rx, and bilingual breastfeeding education and workplace policy), and one strategy to improve community-clinical linkages (a community health worker-led diabetes prevention program). For each program presented in this community case report, we also summarize several activities that we carried out to incorporate the needs and preferences of our Hispanic population in order to make these programs culturally appropriate (e.g., bilingual staff, data collection activities in Spanish and English, marketing campaign with cultural elements, cultural competency training). We also introduce our community-academic partnership and the conceptual model guiding our work.

## Context: community-academic partnership

**Figure 1:**
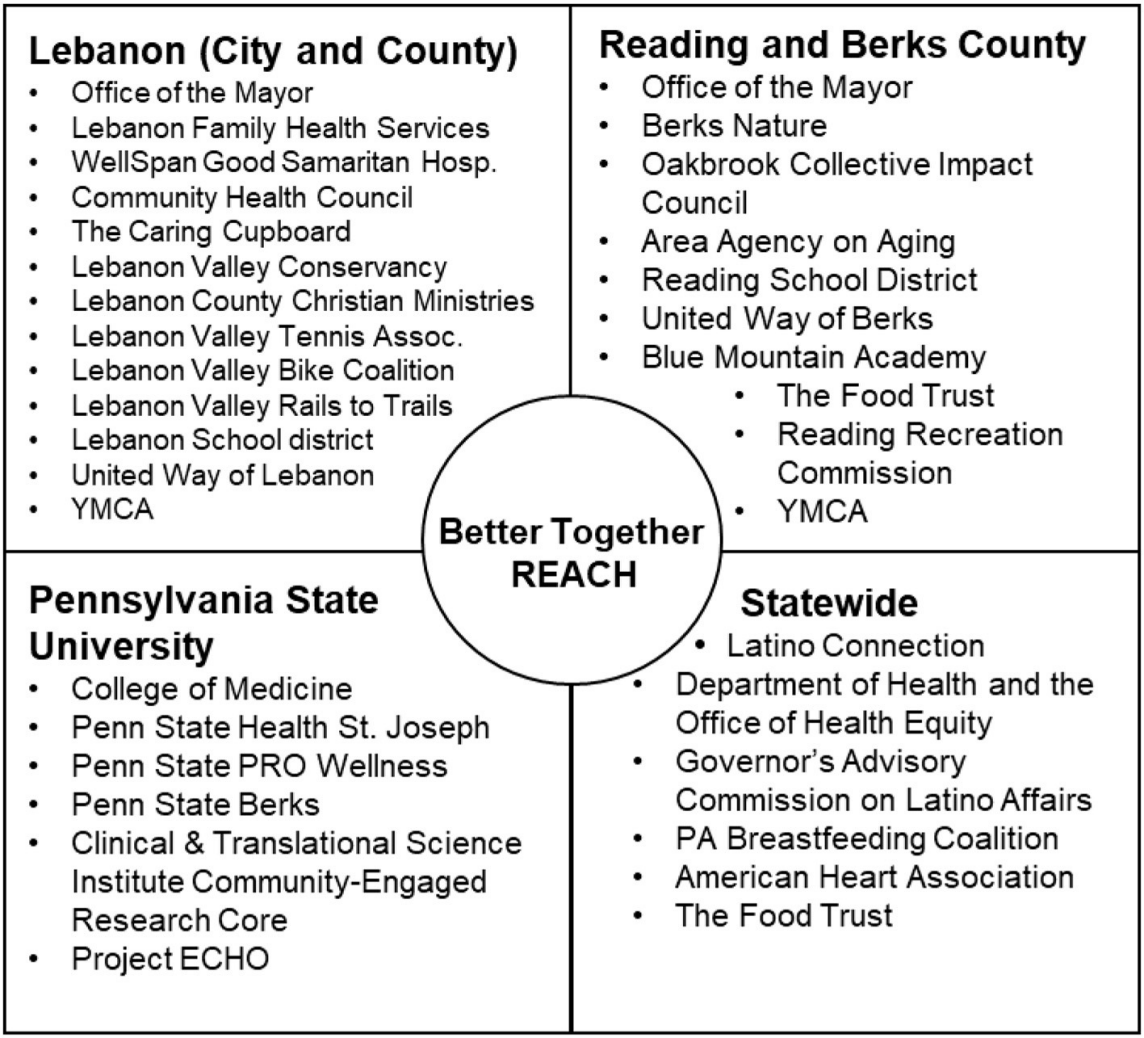
Better Together REACH community-academic partnership.

The Better Together initiative was first conceived on October 2016 when a community health advocate in Lebanon found that many local organizations were offering community health and social services but operating independently. With a longstanding history in prevention and community engagement, Penn State PRO Wellness partnered with these organizations to bring them together in a community–academic partnership. Since then, over 60 local organizations are collaborating to break down silos and share a common agenda: address health disparities, improve the quality of life and wellness of residents, and promote healthier environments where people live, work, learn and play. The original initiative was organized around four action themes, including healthy food access, physical activity, mental and behavioral health, and family and community engagement. These themes were perfectly aligned with the goals of the overall REACH program. Our team leveraged these existing collaborations, shared interests, and the expansion of Penn State Health into Berks County to apply for the REACH award. Lebanon and Berks counties are geographically close and share similar demographics and identified community needs, creating areas for cooperative integration of leading organizations and their programming within these communities. Currently, Better Together REACH benefits from the active involvement of many local and statewide organizations with established connections with the Hispanic community (Figure 1). Convening individuals and leaders from many layers of the community helped to ensure discussions were representative and inclusive of the overall community voice.

## Conceptual model

**Figure 2:**
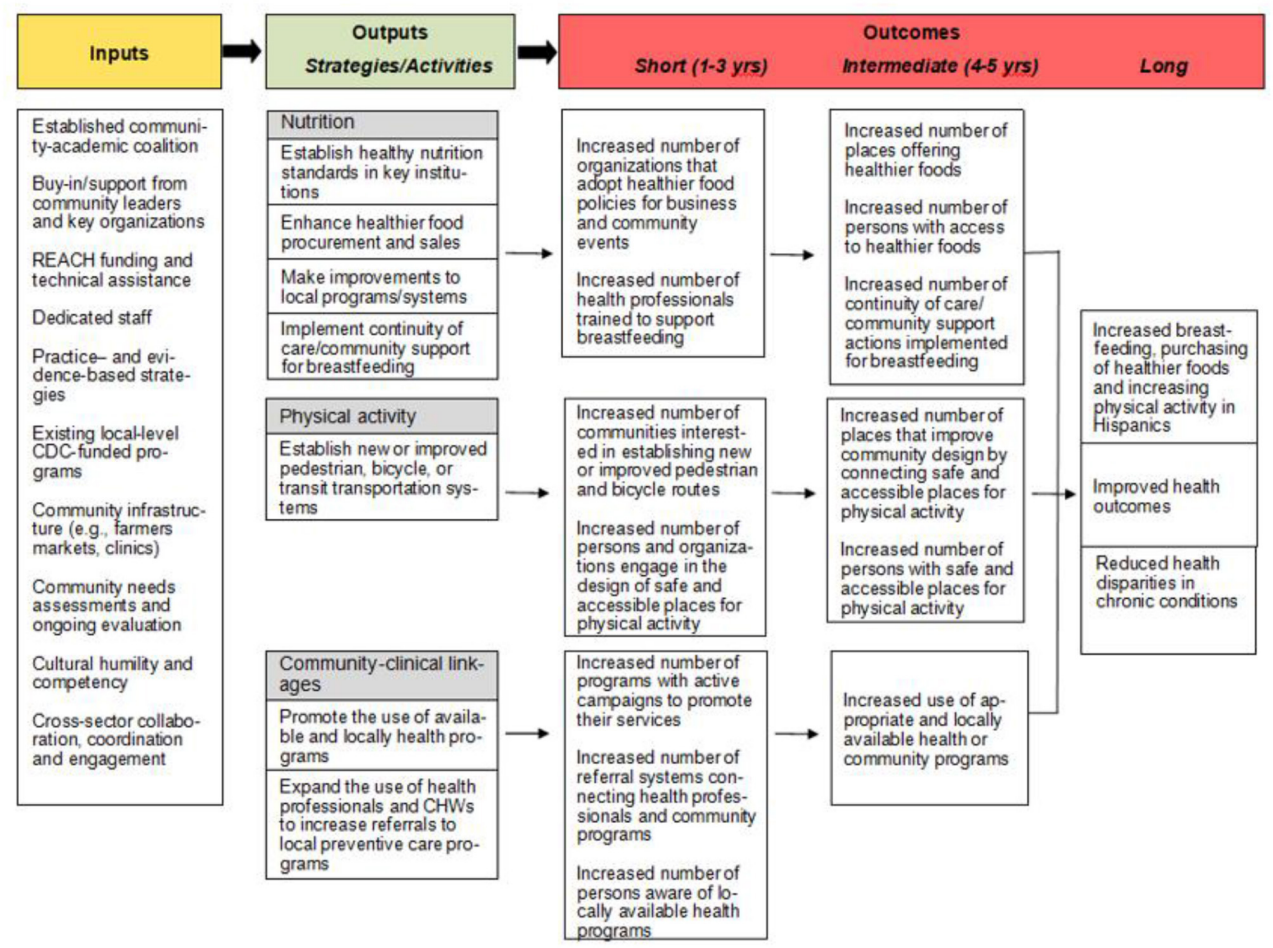
Conceptual model guiding the evaluation of Better Together REACH.

Figure 2 shows our model, which fully incorporates the inputs and activities of Better Together REACH to evaluate the proposed outcomes related to physical activity, nutrition (including breastfeeding), and community-clinical linkages. Importantly, we have identified short, intermediate, and long outcomes that align with the overall REACH objectives, the Healthy People 2020 goals pertaining to chronic disease prevention, our coalition's capacity, and the needs identified from multiple CHNAs. Beyond the project period, our coalition expects to achieve the following long-term outcomes: increased purchasing of healthier foods and increased levels of physical activity (e.g., percentage of Hispanics adults and youth who meet physical activity guidelines), reduced health disparities in chronic conditions and risk factors (e.g., percentage of Hispanic adults and youth who are overweight or obese), and improved health outcomes (e.g., percentage of Hispanic adults with type 2 diabetes).

## Programmatic components

Since 2018, our Better Together REACH partnership has implemented several locally-tailored practice- and evidence-based strategies aimed at increasing physical activity opportunities, healthy nutrition programming, and diabetes prevention programs in Lebanon and Berks counties (Table 2). In this section we describe the work-in-progress and lessons learned from our REACH-supported initiatives.

**Table 2:** Work-in-progress: key programs implemented in years 1–4.

**Program**	**Target population**	**Outcome measure**	**Data source**	**Progress**
City redesigning and master planning	Lebanon County residents	Increase the number of linear miles connecting everyday destinations	Official city documentation, including MOAs	As of fall 2021, 3.32 linear miles have been built or enhanced. Our goal is to reach 11 linear miles by the end of 2022
Interactive resource map	Lebanon and Berks counties' residents	Increase awareness of existing community resources for chronic disease prevention	CHNAs and project surveys	Two interactive maps displaying local resources related to physical activity, healthy eating, DPPs, and social and human services were developed and maintained by our coalition
Nature Rx	Berks County residents	Increase outdoor physical activity	Self-reported via online survey	Our pilot with 38 adults showed that individuals spent 5 days outdoors for ≥10 min and spent an average of 1 hr and 56 min outdoors each week. In a typical week, they spent around 4 days engaging in moderate physical activity with an average of 1 hr and 41 min per week
FMNP	WIC beneficiaries (*n* = 750) who are food insecure in Lebanon County	Increase voucher redemption rates (to purchase fresh vegetables and fruits)	Vouchers prescribed and redeemed, PA Department of Agriculture, Bureau of Food Assistance	In 2019, the FMNP voucher redemption rate was 36.7% and decreased to 25.6% in 2020. A “grab bag” program was implemented in 2021
Veggie Rx	Hispanic prediabetes and diabetic patients (*n* = 326) at the Penn State St. Joseph downtown hospital	Increase consumption of fresh vegetables and fruits; decrease in type 2 diabetes levels	Vouchers prescribed and redeemed, Veggie Rx program	In 2019, 215 patients and family members participated. Patients showed a reduction in HbA1c (−1.3%; *p* < 0.001) post-program. Reduced HbA1c was associated with higher voucher redemption rates (*p* = 0.032) and a change in diabetes medications (*p* = 0.003)
Bilingual breastfeeding education	Expectant or new mothers who receive WIC services at LFHS	Increase the number of women who receive bilingual breastfeeding education	LFHS client log	In 2019 and 2020, 106 and 350 WIC recipients, respectively, received bilingual breastfeeding education. In 2021, all education is being offered online. A major culturally-relevant marketing campaign accompanies the program
DPP	Hispanic prediabetes and diabetic patients in Lebanon and Berks counties	Increase the number of patients with access to bilingual prediabetes and diabetes management education	Penn State Health St. Joseph, LFHS, and Union Community Care (FQHC)	Between 2019 and 2020, 204 patients enrolled in DPP classes offered at these 3 locations

FMNP, Farmers Market Nutrition Program; WIC, Women, Infant, and Children Nutrition Program; CHW, Community health worker; LFHS, Lebanon Family Health Services; MOA, memorandum of Agreements; DPP, Diabetes Prevention Program; CHNAs, Community Health Needs Assessments; FQHC, Federally-qualified Health Center.

## Physical activity

### City redesigning and master planning

We have partnered with the City of Lebanon and the Lebanon Valley Conservancy (a non-profit organization dedicated to the protection, preservation and proper use of natural habitats, historical resources, and farmland) to improve the walkability of the city. One of our first initiatives was the launch of a bilingual mobile app to report issues with the City's streets and recreational areas, including uploading pictures and pin locations. Electronic reports from residents were sent to the City's department of public works for their attention. We also partnered with the Community Health Council of Lebanon to seek WalkWorks designation for a walking trail in downtown Lebanon. WalkWorks is a program run by the Pennsylvania Department of Health and Pennsylvania Downtown Center to recognize local efforts that promote safe walking routes; offer social support through guided, community-based walking groups; help schools develop walk-to-school programs; and address local policies to increase safe walking routes. A 1.32-mile route was awarded WalkWorks designation, providing safe connection to six everyday destinations. This was the first route in Lebanon County with such a designation.

**Figure 3:**
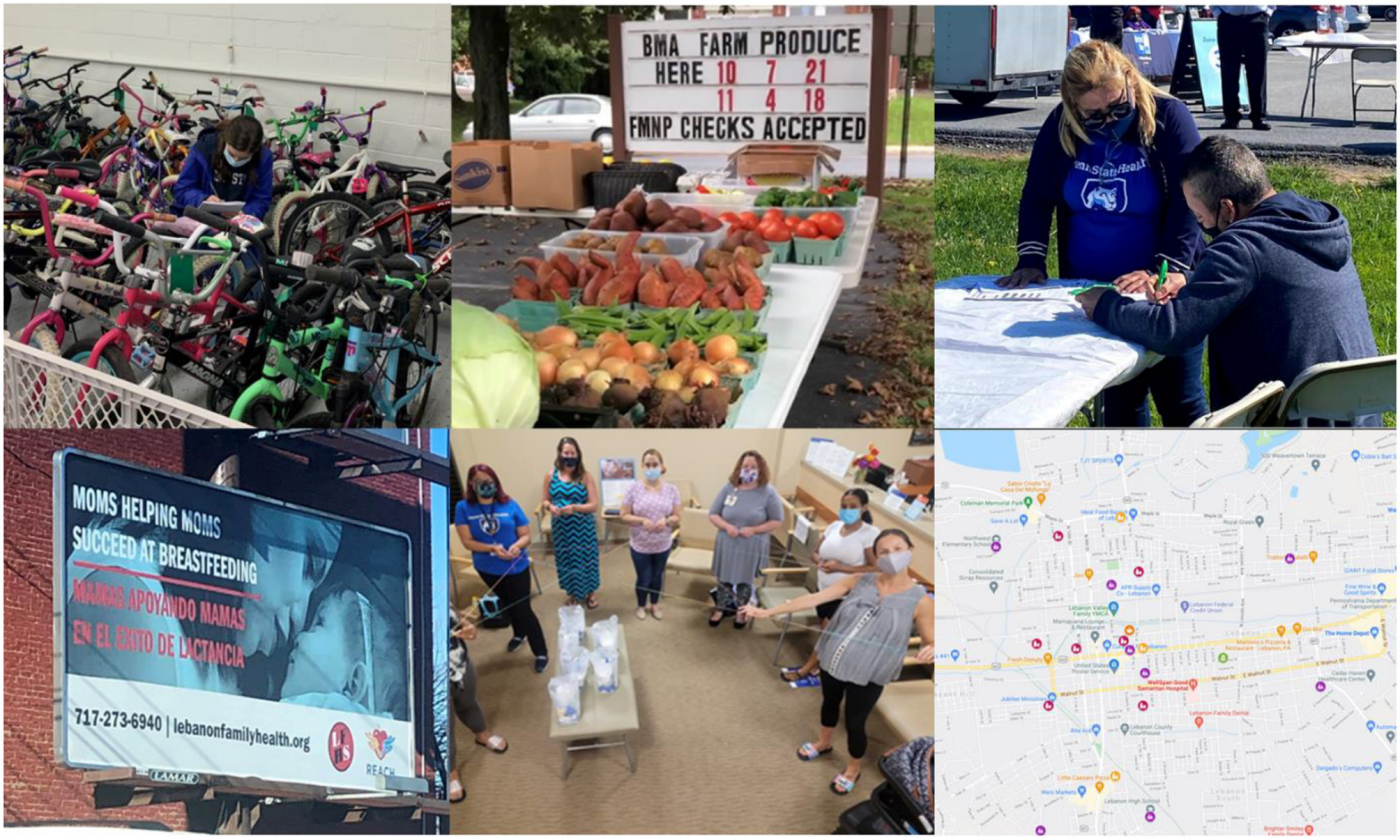
Top row: staff making inventory of repaired bicycles to be distributed to youth and families through the Lebanon Bicycle Recycle **(left)**; farm stand for FMNP and Veggie Rx participants in Reading's Penn State Health St. Joseph hospital **(center)**; and community health worker conducting screenings for diabetes prevention and management programs at a COVID-19 vaccination site in Reading **(right)**. Bottom row: billboard displaying our marketing campaign for breastfeeding in Lebanon **(left)**; bilingual breastfeeding education delivered by a community health worker at a WIC office in Lebanon **(center)**; and interactive map showing local resources for physical activity (e.g., public parks, trails, indoor spaces), healthy eating (e.g., food assistance programs, farmers markets), and diabetes prevention programs in Lebanon **(right)**.

Our team also conducted a survey to assess physical activity practices, use of community green spaces, and specific areas (e.g., recreational areas, sidewalks, streets) that residents would like to see improved. The survey was developed with input from community partners, including Hispanics, and was delivered in Spanish and English to better reach our community. We leveraged these survey results and partnered with the City of Lebanon to help design, enhance, and restructure and urban alley into the Liberty Trail Park, Wengert Memorial Park, and the Borne Learning Trail (part of the Grow Lebanon 2020 plan) through bilingual interactive signage and trail restoration. One pedestrian mile was enhanced and another mile of multi-use trail was created as part of the Borne Learning Trail. The Wengert Memorial Park will serve as a trailhead and connector to 14 miles of the Lebanon Valley Rail Trail. In the park will be a bike safety traffic garden, one of its kind in Pennsylvania, an area for youth to learn traffic rules and practice these skills in a secure and fun environment. Furthermore, we have worked toward installing seven automated eco-counters to monitor pedestrian traffic patterns in Lebanon and Reading. The locations for the measurements were chosen with guidance from community stakeholders to assess the need for new or expanded crosswalks, sidewalks or trails. Our goal is to develop new or enhance existing 11 miles of routes that connect everyday destinations and improve walkability. Our team also developed an interactive map for community members to identify local resources for physical activity (e.g., public parks, trails), indoor spaces where the community can exercise (e.g., YMCA), and other local programming, including emergency food providers, food assistance programs, farmers markets, local orchards, and community gardens (Figure 3).

### Nature Rx

We partnered with Berks Nature, a nonprofit conservation organization, to implement the Nature Rx program to motivate adult and young individuals to spend time outdoors engaging in some sort of physical activity, such as walking, hiking, or biking. We first developed a bilingual marketing campaign to increase awareness of Nature Rx including examples of fun outdoors activities. We also adopted Goose Chase (goosechase.com), a scavenger hunt mobile app available in Spanish and English, to encourage enrollment in Nature Rx and reward participants for spending time outdoors. We invited those who downloaded the app to complete a brief survey to measure their levels of physical activity and engagement with outdoor spaces. Participants reported spending 5 days outdoors for ≥10 min and spent an average of 1 h and 56 min outdoors each week. In a typical week, participants reported spending around 4 days a week engaging in moderate physical activity with an average of 1 h and 41 min per week. On average, participants increased exercise duration by 7 min after completing the program.

### Lebanon Bicycle Recycle

In partnership with the Lebanon Valley Bike Coalition, an organization that works to educate, advocate, and improve physical activity through cycling, we helped launching the LBR in 2021. Meetings with local leaders confirmed that cycling is important in Lebanon for physical activity and transportation to work and school. LBR is a program that collects used bikes, repairs them, and returns them back to community members who need them. To date, over 125 bikes have been given to community members with an emphasis on youth and families (Figure 3). Further, LBR has worked with volunteers to train them to repair bikes, including a cohort of middle-school students who came after school to receive training. Over 280 hours of volunteer time have been given to the program. Also, LBR has partnered with the Migrant Education Program and the Lebanon County Christian Ministries (both organizations serve a large number of Hispanics) to provide bikes to their members to help with their local transportation needs. For instance, we developed a bilingual marketing campaign to promote this program and education to encourage physical activity through cycling, including how to do it safely in the city. Our REACH team is conducting a survey with bike recipients to learn about their levels of physical activity and social needs.

## Healthy eating

### Farmers Market Nutrition Program

The Pennsylvania Department of Agriculture provides those who are eligible four vouchers of six dollars each to spend from June through November on seasonal fruits and vegetables grown by Pennsylvania farmers. Distribution and redemption rates of these FMNP vouchers are lower in Lebanon when compared to neighboring counties. Lebanon Family Health Services (LFHS), a local Women, Infants, and Children (WIC) office had a 36.7% redemption rate of vouchers in 2019; that number dropped to 25.6% in 2020 as a result of the pandemic. The goal of our FMNP initiative is to increase voucher redemption rates among WIC beneficiaries served by LFHS and senior residents. To promote FMNP awareness and voucher redemption, LFHS holds the Latino Health Fair, a one-day annual event that includes live demonstrations on healthy cooking and bilingual nutrition education. In 2019, we conducted a brief evaluation with WIC beneficiaries to survey barriers participants faced in using FMNP vouchers like transportation to voucher-eligible markets, variety of produce, and limited locations of voucher-eligible markets (16). The survey also gauged the participants' interest in additional offerings to improve voucher redemption such as healthy cooking or nutrition education. To adhere to COVID-19 safety protocols, our FMNP initiative was adapted into a “grab bag” program to limit physical contact between participating families and staff while offering the same quality produce in convenient packaging. Informed by our 2019 survey, healthy recipes for cooking with seasonal fruits and vegetables were included in grab bags in both English and Spanish. These recipes included common Hispanic dishes. To further evaluate this new initiative, in fall 2021, we started inviting FNMP participants to complete a brief survey within 24 h of purchasing the grab bag and a second survey was sent within 7 days. Also in 2021, we started evaluating FMNP voucher redemption among seniors in Lebanon to identify potential barriers and areas of opportunity to increase redemption rates and ultimately, the consumption of fresh produce among this vulnerable group. One of the indentified barriers to voucher redemption was low access to vendors who participate in FMNP. In 2022, we worked with a new farmer to obtain the registration as FMNP vendor and we then partnered with the Chestnut Street Community Center (CSCC) in downtown Lebanon to host a new farmers market. CSCC is the location for two Hispanic churches, two non-profit organizations, an overnight shelter, and a new community garden. This location is ideal for expanding FMNP as it is along a popular bus route and walking distance to a WIC office and a local elementary school. Importantly, this new farmers market is the only one in the City of Lebanon that accepts FMNP vouchers provided to both WIC and senior participants.

### Veggie Rx

Veggie Rx is a fruit and vegetable prescription program that allows clinical staff at Penn State Health St. Joseph in Reading to prescribe vouchers for fresh produce to patients with chronic, diet-related health conditions (17). The program was launched in collaboration with the United Way of Berks and The Food Trust. The goal of Veggie Rx is to increase access to and consumption of local fresh produce among patients with diabetes. Clinic providers prescribe produce vouchers ($28-$140/month), to Supplemental Nutrition Assistance Program (SNAP)-eligible patients based on their patients' household size, that can be redeemed at local farmers markets and participating retailers. During 6-month follow-up and group education sessions with a dietitian, patients fill out a nutritional assessment for daily fruit and vegetable consumption and discuss situations that may have changed their nutritional habits. To improve opportunities to redeem vouchers, a farm stand by a local farmer is located within the St. Joseph's downtown campus. This initiative is also assisted by one of our REACH-funded bilingual community health workers (CHWs). A pilot study with 97 adults with type 2 diabetes [hemoglobin A1c (HbA1c) ≥ 7.0% and body mass index (BMI) ≥ 25 kg/m^2^] showed a reduction in HbA1c (-1.3%; *p* < 0.001) post-program (16). Reduced HbA1c was associated with higher voucher redemption rates (*p* = 0.032) and a change in diabetes medications (*p* = 0.003). There were no associations with BMI, but changes in blood pressure was positively associated with Veggie Rx voucher redemption. Given the initial success of Veggie Rx in Reading, our team is expanding this initiative to Lebanon in partnership with Union Community Care (UCC), a local federally-qualified health center. We anticipate enrolling 30 UCC patients into the Veggie Rx program in the next year.

### Bilingual breastfeeding education and workplace breastfeeding policy

We partnered with LFHS, the local WIC provider in Lebanon, to provide bilingual breastfeeding support to expectant or new mothers. With REACH funds we hired a full-time CHW and trained her in the provision of professional lactation care. Since 2019, our CHW has provided one-to-one and group education to 456 pregnant and postpartum women at the WIC clinic, local hospitals, or by phone in both Spanish and English. To abide by COVID-19 protocols and guarantee the safety of our CHW and WIC recipients, all counseling sessions were moved online. To better support this initiative, our REACH program developed and disseminated a culturally-relevant marketing campaign about breastfeeding support across local healthcare organizations and the community, including media advertising in billboards and public transit (Figure 3). Partnering with the Lebanon Valley Chamber of Commerce, we also conducted a survey to determine what breastfeeding policies are in place at Lebanon County's businesses. Our team also conducted a series of focus groups with women who have returned to work while breastfeeding to learn how they are or are not supported through workplace policies. We found that polices supporting breastfeeding in workplaces were not consistently implemented.

## Community-clinical linkages

### Community health worker-delivered diabetes prevention program

We partnered with the Langan Allied Health Academy-CHW Training Institute to recruit and train bilingual CHWs for work in community and outpatient/inpatient settings. To date, the Institute has trained 11 class cohorts and over 120 CHWs. The training provides comprehensive education in 15 areas including cultural competency, health literacy, patient navigation, and chronic disease prevention and management. Our project hired three local bilingual CHWs to conduct diabetes screenings at community events and provide DPP classes. Our CHWs completed their training with the Institute as well as a CDC-certified lifestyle coach training. At present, our CHWs have provided CDC-approved DPP to 204 residents at three clinical facilities in Lebanon and Berks counties. We have worked with LFHS to provide bilingual DPP in Lebanon. Serving two cohorts a year, 60 people have participated in this program. In 2022, REACH also supported the offering of DPP at Union Community Care (UCC), a federally qualified health center in Lebanon. We also supported the CHW at Family First Health, another federally qualified health center in Lebanon, to become a certified lifestyle coach in order to offer CDC-approved DPP. Each of these locations, LFHS, UCC and Family First, offers bilingual DPP classes, including in-person and Zoom options.

## Discussion

Better Together REACH has made significant progress in addressing health disparities related to chronic disease prevention in the Hispanic-majority communities of Lebanon and Reading. Of note, we are actively promoting existing walking and bike routes that connect everyday destinations and supporting the planning and designation of new routes (e.g., WalkWorks, Born Learning Trail), which are evidence-based strategies to improve physical activity (18). We are also expanding access to affordable and nutritious food (e.g., FMNP, Veggie Rx) to alleviate food insecurity among low-income families and creating bilingual breastfeeding education programming to support WIC beneficiaries in their preferred language. To address critical community-clinical linkages, we are expanding access and referrals to DPP offerings by training local, bilingual CHWs to connect at-risk individuals with new and existing programs. CHWs are trusted voices in the community who can support community-clinical initiatives for chronic diseases (19, 20). Our initiatives are promoted throughout strong community networks with culturally-relevant marketing campaigns. A good number of our annual objectives are being met but much work lies ahead as we recover from the pandemic (21). The pandemic impacted our coalition's ability to deliver activities as many were planned as in-person events or large community gatherings. However, the limitations created by the pandemic offered opportunities to innovate, evidenced by our grab bag program and the virtual breastfeeding education. Other REACH programs can employ similar approaches to support their priority populations with needed chronic diseases prevention programming during these challenging times.

Through our established community-academic partnership, we will continue implementing locally-tailored practice- and evidence-based strategies to address chronic disease disparities in Lebanon and Reading. These strategies were also tailored to respond to the cultural preferences and needs of Hispanics in the two target communities. Our next program evaluations will incorporate data from novel techniques including automated eco-counters to monitor pedestrian traffic patterns. This approach is promising because it incorporates easy-to-use technology into community-level interventions and involves community members in making decisions about where to create new or expand trails. This level of community engagement and shared decision-making is critical to meet our programmatic goals related to physical activity, including the creation of a bicycle and safety garden and mobile bicycle repair unit, as well as a park assessment initiative. Future program evaluation data will also be collected from new initiatives impacting nutrition and breastfeeding goals. For example, in collaboration with the local Chamber of Commerce we will champion a “Breastfeeding is Welcome Here” campaign among Lebanon employers after assessing existing policies and practices. Findings will inform the creation of resources and training to support breastfeeding practices at local worksites. We will also increase the number of worksites with implemented health nutrition standards by working with local food pantries to develop and evaluate placement and behavioral design initiatives.

Our team has always been looking for new community partners to innovate our programming. For example, with the lessons we have learned in the past 4 years, we look forward to expanding our DPP beyond healthcare settings and directly into local employers. We have partnered with the largest poultry producer in the region to offer an online version of DPP for their employers. Almost 90% of the employees of this company are Hispanics, have limited English proficiency, and reside in the counties of Lebanon and Berks. This partnership will offer expanded health services to those who need them most. If this online DPP is successful, it would be replicated with other employers in our catchment area. Also, our team is continuing to refine programmatic data collection and measurements, including using geographical information mapping to evaluate our activities and provide timely feedback to our community partners. We will continue monitoring our coalition's capacity and updated CHNAs to revise our projected outcomes, but always aligned with practical strategies for culturally competent evaluation (22). Although not considered in the present project, there are several promising chronic disease prevention and management interventions that others working with Hispanic populations could consider implementing. For example, employer-based walking challenge campaigns, (23) obesity reduction education at community venues (e.g., senior centers, churches, low-income housing, community centers), (24) and CHW-led physical activity programs (25). These interventions have shown broad reach of Hispanic individuals, good implementation feasibility, and effectiveness to improve physical activity and reduce obesity (23–25).

The national goal of achieving health equity cannot succeed without eliminating Hispanic health disparities and engaging communities to seek and disseminate culturally-relevant solutions. Together with its partners, Better Together REACH is working toward that goal and on capacity building with the aim of continuity and sustainability of this initiative post-award. Intervening in areas facing high chronic disease health disparities, like Lebanon and Reading, leads us to develop a community-collaborative blueprint that can be replicated across Hispanic communities in Pennsylvania and the United States.

## Data availability statement

The raw data supporting the conclusions of this article will be made available by the authors, without undue reservation.

## Ethics statement

The studies involving human participants were reviewed and approved by the Penn State Institutional Review Board. The patients/participants provided their written informed consent to participate in this study. Written informed consent was obtained from the individual(s) for the publication of any potentially identifiable images or data included in this article.

## Author contributions

WC led the development of the manuscript. WC, BA, AM, LC, and JK led the development of the data collection instruments. All authors contributed to the data acquisition, analysis, interpretation, made substantial contributions to this manuscript, and approved the version submitted.
